# Clinical and Pathological Features of Childhood-Onset Nemaline Myopathy: A Report of Four Cases

**DOI:** 10.1155/2012/203602

**Published:** 2012-07-31

**Authors:** Chao Jiang, Jianping Wang, Haidong Lu

**Affiliations:** ^1^Department of Neurology, The Fifth Affiliated Hospital of Zhengzhou University, Henan, Zhengzhou 450052, China; ^2^Department of Neurology, The People's Hospital of Jiaozuo City, Henan, Jiaozou 450052, China

## Abstract

We examined whether immunological abnormalities can be found in the specimens of four childhood-onset nemaline myopathy (NM) patients without autoimmune diseases. Pathological examination revealed that nemaline rods were found in all specimens. The immunohistochemical results showed that CD4 positive cells and some other cells were gathered among the necrotic muscle fibers. We conclude that immunological abnormalities are present in the specimens of certain childhood-onset NM patients without autoimmune diseases. Further evaluation of the immunological changes is warranted in childhood-onset NM patients.

## 1. Introduction

The association between autoimmune diseases and nemaline myopathy (NM) has always been reported. Research showed that immunosuppressive treatment is effective to adult-onset NM, which suggests that NM may also be a kind of dysimmune origin disease [1–3]. However, little is known about the immunological changes in the specimens from childhood-onset NM patients without autoimmune diseases. Here, we described the clinical and pathological features of four cases of childhood-onset NM patients without autoimmune diseases and examined whether immunological abnormalities can be found in their specimens.

## 2. Case Reports

Four patients, one female and three males, with an age range of 6–12 years old (mean age 9 years old) were admitted to our hospital. The clinical manifestations were as follows: three of them with progressive hypotonia of both lower extremities, whereas another case with progressive hypotonia of limbs. The respiratory function of the four patients is normal. Physical examination revealed that the vital signs, development, consciousness, and intelligence, are normal. There is no atrophy of both sternocleidomastoid muscles. The following lab tests are normal: erythrocyte sedimentation, blood lipid, hepatic function, antistreptolysin O, C reaction protein, antinuclear antibody, immunoglobulin, antibody of human immunodeficiency virus (HIV), and syphilis. Abdominal ultrasound, ECG, color Doppler ultrasound, and cardiac output showed negative results. The creatine phosphokinase (CPK) level in one of the four patients was higher than that in normal controls. Myogenic damage of affected muscle was examined by electromyography. The four patients did not have family medical history or other immune diseases. Results from clinical, laboratory, and ancillary investigations of the four patients are summarized in Table 1.

## 3. Pathological Findings

All muscle biopsies had been received in a fresh state from vastus lateralis of left quadriceps and were divided into two portions. One portion was quickly frozen in liquid nitrogen cooling isopentane, and 8 *μ*m frozen sections were cut and stained with hematoxylin and eosin (H and E), modified Gomori trichrome (MGT), periodic acid Schiff (PAS), oil red O, ATPase at pH 9.6, 4.6, and 4.3, nicotinamide adenine dinucleotide-tetrazolium reductase (NADH-TR), succinic dehydrogenase (SDH) alone as well as combined with cytochrome oxidase (Cox with SDH), myophosphorylase, amylopectinase phosphofructokinase, adenylate deaminase. HE, MGT, and ATPase stains are essential for the diagnosis and differential diagnosis of muscle diseases; PAS, oil red O, NADH-TR, SDH, COX, myophosphorylase, amylopectinase phosphofructokinase, and adenylate deaminase staining were used to exclude glycogen storage diseases, mitochondrial myopathy, lipid storage myopathy, and so on. In addition, CD4 and CD8 immunostaining was used to examine immunological abnormalities in the specimens. Another part of specimens was fixed in the 2% glutaraldehyde and embedded with Epon812, 1 *μ*m semithin slices were then cut for toluidine blue stain and made into thin slices for lead-uranium double stain, and myopathic features were detected on electromyography (Hitachi H2800).

**Figure 1:**
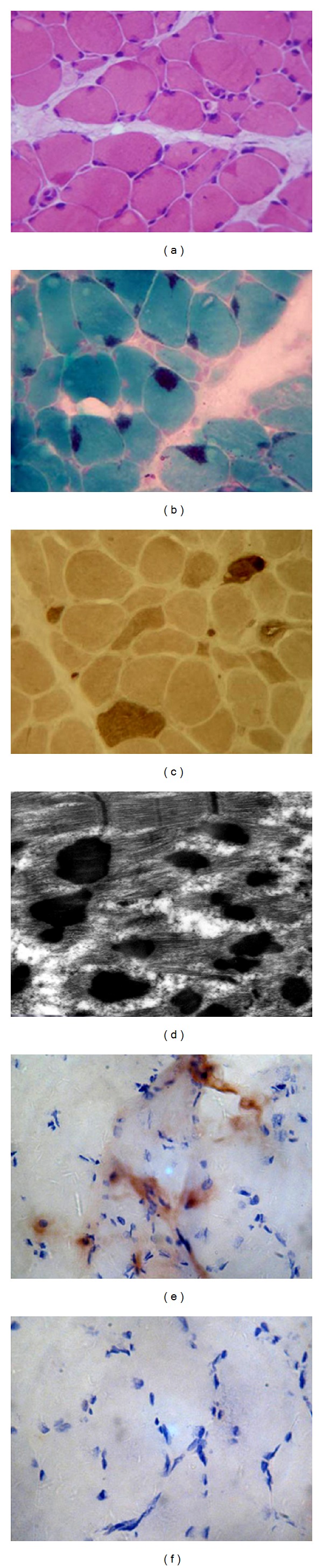
(a) HE staining revealed inequality of size of muscle fibers and a lot of emarcid muscle fibers and compensatory hypertrophic muscle fibers. Some dark red uniform of eosinophils matters was observed among some of the muscle fibers. Magnification ×400. (b) Modified Gomori trichrome (MGT) staining showed dark blue rod-like structures or nemaline rods, and abnormal red fiber was not found. Magnification ×400. (c) Histochemistry of ATP enzyme staining demonstrated abnormal distribution of type I (predominated) and type II (rare) muscle fibers. Magnification ×400. (d) Lead-uranium double staining demonstrated myofibril disorderly arrayed, and a large number of nemaline body under the sarcolemma and between the myofibril, and electron dense nemaline bodies of Z-band origin on ultrastructural examination. Magnification ×12000. (e), (f) Immunohistochemical results of the two specimens with necrotic muscle fibers showed that CD4 positive cells and some other cells were gathered among the muscle fibers (e), but CD8 positive cells were not found (f). Magnification ×400.

 HE staining revealed inequality of size of muscle fibers and a lot of atrophic muscle fibers and compensatory hypertrophic muscle fibers in all cases. In addition, some dark red uniform of eosinophilic matters was also observed between some of the muscle fibers (Figure 1(a)). Dark blue rod-like structures or nemaline rods were observed under light microscopy with MGT stain, whereas abnormal red fibers were not found (Figure 1(b)). Histochemistry of ATP enzyme staining demonstrated Type I fiber predominance in all cases of NM (Figure 1(c)). However, we did not find any evidence of positive results when stained with oil red O or PAS. Necrotic muscle fibers were found in two specimens, and the immunohistochemical results of these two specimens showed that some CD4 positive cells and other cells were gathered among the muscle fibers (Figure 1(e)), but CD8 positive cells were not found (Figure 1(f)). Electron microscopic examination revealed characteristic rod-like structures were observed predominantly in the subsarcolemmal location but not within the myofibers, and their morphology varied from single rod to large aggregates. These rods appeared as lattice-like structures similar to Z-band material under higher magnification. No intranuclear rods were found in any case (Figure 1(d)).

## 4.   Discussions 

In this paper, we reviewed the clinical materials of four cases with childhood-onset NM. Although previous reports revealed that some of these patients present with dilated cardiomyopathy [4] or hypertrophic cardiomyopathy in children [5], the associated cardiac involvement was not found in these four patients. MGT staining and electron microscopic examination demonstrated that the nemaline body existed within the normal size muscle fibers and atrophic muscle fibers. Muscle fibers with nemaline body account more than 60 percent of the total fibers, and the proportion in each muscle bundle varied, which is similar to what is described in a previous report; the proportion of muscle fibers with nemaline body correlated with the time course of disease progress but did not correlate with the age of onset and the severity of disease [6].

The cause of NM is not clear. Previous studies revealed that childhood onset nemaline myopathy may be a genetic condition with at least six different causative genes [7]. Other research found that about half of the late onset NM patients complicated with immune system diseases, such as HIV, human T-cell lymphocytes infected with the virus, monoclonal gammopathy, and primary hypothyroidism [8, 9]. However, the association between childhood onset NM and immunological abnormalities was rarely reported. In this paper, we found that necrotic muscle fibers were found in two specimens of the four cases. In addition, immunohistochemical results revealed that some CD4 positive cells and other cells were gathered among the muscle fibers. We concluded that inflammatory cell infiltration may be associated with the necrosis of some fibers, and immunological abnormalities were found in some childhood-onset NM patients without autoimmune diseases. Although we agree that childhood onset NM is a genetic muscular disorder, the immunological abnormalities may be also a characteristic feature in some cases. To clarify its association with abnormal gene expression, and which gene may play a role in this pathologic process, further study is warranted. 

## Figures and Tables

**Table 1:** Results from clinical, laboratory, and ancillary investigations of the four patients (the normal value of CPK should be less than 183 U/L).

Age/sex	Onset age	Family history	CPK	Seroimmunity	EMG	ECG
6/M	3 years	—	69 U/L	—	Myopathic	Normal
8/F	4.5 years	—	113 U/L	—	Myopathic	Normal
10/M	6 years	—	84 U/L	—	Myopathic	Normal
12/M	10 years	—	201 U/L	—	Myopathic	Normal

## References

[1] Shy GM, Engel WK, Somers JE, Wanko T (1963). Nemaline myopathy: a new congenital myopathy. *Brain*.

[2] Ryan MM, Schnell C, Strickland CD (2001). Nemaline myopathy: a clinical study of 143 cases. *Annals of Neurology*.

[3] Engel WK (2007). Late-onset rod myopathy with monoclonal immunoglobulin can be treatable with IVIG. *Abstracts/Neuromuscular Disorders*.

[4] Müller-Höcker J, Schäfer S, Mendel B, Lochmüller H, Pongratz D (2000). Nemaline cardiomyopathy in a young adult: an ultraimmunohistochemical study and review of the literature. *Ultrastructural Pathology*.

[5] Sharma MC, Gulati S, Atri S (2007). Nemaline rod myopathy: a rare form of myopathy. *Neurology India*.

[6] Ryan MM, Ilkovski B, Strickland CD (2003). Clinical course correlates poorly with muscle pathology in nemaline myopathy. *Neurology*.

[7] Goebel HH (2003). Congenital myopathies at their molecular dawning. *Muscle and Nerve*.

[8] Mani D, Aboulafia DM (2008). Editorial comment: HIV-associated adult-onset nemaline myopathy. *AIDS Reader*.

[9] Keller CE, Hays AP, Rowland LP, Moghadaszadeh B, Beggs AH, Bhagat G (2006). Adult-onset nemaline myopathy and monoclonal gammopathy. *Archives of Neurology*.

